# Internal consistency, construct validity, and responsiveness of the MRC Prion Disease Rating Scale

**DOI:** 10.1186/s41687-025-00884-3

**Published:** 2025-05-06

**Authors:** Leah Leidy, Aaron Yarlas, Robert S. Pulido, Jessica Ludwig, Kathleen Glisic, Brian S. Appleby

**Affiliations:** 1 Departments of Neurology and Psychiatry, Case Western Reserve University/University Hospitals Cleveland Medical Center, Cleveland, OH USA; 2 Department of Pathology, Case Western Reserve University/University Hospitals Cleveland Medical Center, Cleveland, OH USA; 3 National Prion Disease Pathology Surveillance Center, Department of Pathology, Case Western Reserve University, Cleveland, OH USA; 4 Florida Atlantic University Charles E. Schmidt College of Medicine, 777 Glades Road BC-71, Boca Raton, FL 33431 USA; 5 Ionis, Carlsbad, CA USA

**Keywords:** Prion disease, Creutzfeldt-Jakob disease, MRC Prion Disease Rating Scale, Psychometric validation

## Abstract

**Background:**

The Medical Research Council-Prion Disease Rating Scale (MRC-PDRS) is a 20-point clinician-reported outcome scale to assess disease progression in patients with prion disease, an invariably fatal neurodegenerative disease caused by misfolded prion protein. This study aims to evaluate the measurement properties and interpretability of the MRC-PDRS to support the measure’s use for effective disease management and research evaluating effectiveness of treatment options for prion diseases.

**Methodology:**

Utilizing patient data from the Telemedicine Assessment Program for CJD (TAPCJD), statistical assessment was conducted of internal consistency, construct validity (including convergent, divergent validity, and known-groups discriminant validity), responsiveness, and interpretation guidelines using distribution-based approaches to estimate thresholds indicating minimal important change (MIC) in MRC-PDRS scores. Criterion measures used for evaluating construct validity and responsiveness included the Telephone Interview for Cognitive Status (TICS) and Neuropsychiatric Inventory–Questionnaire (NPI-Q).

**Results/Conclusions:**

These findings provide strong preliminary evidence that the MRC-PDRS is reliable, valid, and responsive as a tool for measuring disease progression in patients with prion disease, with preliminary MIC estimates ranging from 1 to 3 points. This supports the use of MRC-PDRS in evaluating potential treatment benefits of prion disease clinical trials, and potentially in clinical practice settings.

**Supplementary information:**

The online version contains supplementary material available at 10.1186/s41687-025-00884-3.

## Background

Human prion diseases constitute a group of neurodegenerative diseases characterized by the misfolding of the cellular prion protein (PrP^C^) into its pathological form, typically referred to as scrapie prion protein (PrP^Sc^) [1]. Presently, prion diseases are universally fatal without any treatment options. The etiology of prion diseases can be categorized as sporadic (sporadic CJD, variably protease sensitive prionopathy, sporadic fatal insomnia), genetic (genetic CJD, Gerstmann-Straussler-Scheinker disease, fatal familial insomnia), or acquired (kuru, iatrogenic CJD, variant CJD) [2]. Sporadic prion diseases are the most common consisting of 85% of cases, followed by genetic making up 10–15% of cases, and acquired forms make up < 1% of cases. Sporadic Creutzfeldt-Jakob disease (sCJD), the most common prion disease, commonly presents with non-specific neurodegenerative symptoms which may mimic other illnesses. As such, sCJD is often misdiagnosed until later stages of the disease, when more typical symptoms specific to sCJD, such as myoclonus, appear [3, 4]. Confirmation of prion disease is definitively made by neuropathologic examination at the time of autopsy. The mean age of patients diagnosed with sCJD is 67 and the prognosis of sCJD is poor, with an average survival of four to six months following symptom onset [5]. It is not uncommon for patients to progress from mild impairment to distinct neurodegenerative symptoms and death within a matter of weeks to months; in 85–90% of sCJD cases, patients die within one year [6, 7].

The Medical Research Council (MRC) Prion Disease Rating Scale (MRC-PDRS) is a clinician-rated outcome instrument originally developed using elements from the three commonly used rating scales in neurologically progressive diseases: the Barthel Activities of Daily Living Index, the Clinical Dementia Rating Sum of Boxes, and the Glasgow Coma Score [8]. The MRC-PDRS was developed for use in prion disease and is structured to assess disease severity and progression over time. It is not meant to be used to assist in diagnosis or differentiating between other conditions. Previous clinical trials in prion disease primarily used survival time as a primary outcome measure [9–16]. However, prion disease is a rapidly progressive neurologic disease that causes significant disability. A scale that can capture disease severity accurately could be used to determine the effectiveness of an experimental treatment that address disability and quality of life concerns as well as accounts for variability in survival times observed between different subtypes of prion disease.

The MRC-PDRS is largely an assessment of an individual’s daily functioning and includes questions regarding continence, mobility, ability to attend to activities of daily living, communication, and cognitive abilities. While findings from the existing literature provide support of the inter-rater reliability and construct validity of the MRC-PDRS [8, 17], there is a lack of available evidence addressing other measurement properties of the instrument, including its internal consistency, responsiveness (i.e., ability to detect change over time), or interpretation guidelines, such as thresholds that would reflect a minimally important change (MIC) in a patient’s MRC-PDRS score. In clinical trials, responsiveness is a key component in the ability to detect changes in a patient’s condition over time to evaluate treatment benefit, and similarly in patient management by allowing healthcare providers a tool to interpret changes in health status.

This study aims to evaluate the internal consistency, construct validity, and responsiveness of the MRC-PDRS utilizing data from participants enrolled in the Teleneurology Assessment Program for CJD (TAPCJD) using scores from other instruments administered in the same patients in this non-interventional, longitudinal study to examine the latter two measurement properties [18]. Additionally, to aid in interpretation of changes in MRC-PDRS scores, distribution-based analyses were conducted to estimate thresholds indicative of minimally important change.

## Methods

One hundred and one (101) TAPCJD participants were included in these analyses. TAPCJD participants met criteria for probable or definite prion disease per standardized diagnostic criteria [19]. TAPCJD is a longitudinal natural history study conducted remotely that collects standardized clinical assessments, including MRC-PDRS (measuring functional status), Telephone Interview for Cognitive Status (TICS; measuring cognitive impairment), and Neuropsychiatric Inventory Questionnaire (NPI-Q; measuring neuropsychiatric symptoms) scales, at all study visits [18]. These data underwent statistical analyses to evaluate internal consistency, construct validity, and responsiveness of the MRC-PDRS. An interim analysis based on the 101 patients who have died was conducted for the purposes of this study. Autopsies were performed through the National Prion Disease Pathology Surveillance Center (NPDPSC) (http://www.cjdsurveillance.com) to confirm definite prion disease diagnosis. TAPCJD and NPDPSC study protocols were approved by the University Hospitals Cleveland Medical Center Institutional Review Board.

The use of these assessments for the TAPCJD study have already been described previously [18], where physicians familiar with prion disease performed the standardized assessments on the research participants. A standardized history was taken from the participant and a study partner that included information on symptom progression, social history, family history, neuropsychiatric history, and medical history. Illness duration is defined as the time from initial symptom(s) to death. Initial symptom(s) were defined as the first consistent and persistent symptom(s) that could be attributed to the disease. Following an initial assessment, two monthly follow-up visits were conducted, with following visits every other month for two visits, followed by visits every three months up until the patient died or withdrew from the study [18]. The scheduling interval aimed to have a window of no more than seven days before or following the expected visit interval.

### Assessments

#### MRC-PDRS

Daily functional ability was measured using the MRC-PDRS, for which a clinician interviews the study partner and the patient (if the patient is capable of communicating accurate information) to inform their assessment. Scores range from 0–20, with higher scores denoting better functioning. The score is calculated as the raw sum of each individual domain score. The domains and their scoring ranges include bowel function (0–1), bladder function (0–1), toilet use (0–2), bathing (0–2), feeding (0–2), transfer and mobility (0–2), stairs (0–2), best verbal response (0–4), memory and orientation to surroundings (0–3), judgment and problem solving (0–1), and use of tools (0–1) [8].

#### TICS

Cognitive impairment was measured using the Telephone Interview for Cognitive Status (TICS) version from Brandt et al [20]. The highest possible score is 41. Patients with scores ≥ 33 are classified as “not impaired”; those with score ranging from 26 to 32 (inclusive) are classified as “ambiguous impairment”; those with scores ranging from 21 to 25 (inclusive) are classified as “mildly impaired”; and those with scores ≤ 20 are classified as “moderately to severely impaired.” Points on the assessment are granted for correct answers provided by the participant. Example questions on the assessment include asking the participant to state their full name, date, and current location, in addition to questions requiring recall of a list of 10 words and performative commands such as tapping the telephone microphone five times [20].

#### NPI-Q

Neuropsychiatric symptoms were measured using the NPI-Q, an observer-rated instrument which consists of 12 neuropsychiatric domains, and for those endorsed, the severity and caregiver distress scores resulting from those symptoms. Symptoms which can be endorsed on the NPI-Q include delusions, hallucinations, agitation/aggression, depression/dysphoria, anxiety, elation/euphoria, apathy/indifference, disinhibition, irritability/lability, aberrant motor behavior, sleep and nighttime behavior disorders, and appetite and eating disorders. The number of symptoms endorsed is tallied. For each symptom endorsed, symptom severity for the patient is rated on a 3-point scale with 1 = mild, 2 = moderate, and 3 = severe, while caregiver distress is scored on a 6-point scale with 0 = not distressing at all, 1 = minimal distress, 2 = mild distress, 3 = moderate, 4 = severe, and 5 = extreme or very severe. Higher scores on NPI-Q scale indicate a higher number of symptoms, degree of severity, and caregiver distress amidst the symptoms, with possible ranges for each score being of 0 to 12 for number of symptoms endorsed, 0 to 36 for symptom severity, and 0 to 60 for caregiver distress [21].

### Analyses

The purpose of the current analyses was to evaluate internal consistency, construct validity, and responsiveness of MRC-PDRS, as well as to estimate thresholds representing minimally important change (MIC) to aid in interpretation of MRC-PDRS scores.

#### Internal consistency

Internal consistency provides an index of the reliability of a scale by evaluating the degree to which items are unidimensional, that is, measuring the same underlying construct. A scale with a high degree of internal consistency indicates the items are strongly intercorrelated and thus reliably assess the same characteristic. To assess internal consistency, we evaluated Cronbach’s α, as well as McDonald’s ω, the latter of which does not assume tau-equivalence (i.e., that factor loadings of all items on the latent construct are equal), which can lead to an underestimation bias of the reliability estimate by Cronbach’s α [22]. A value of at least 0.70 is indicative of adequate internal consistency [23]. Internal consistency was evaluated at each of the first four visits for each patient, all of which had samples including at least 25 patients, which was considered to be sufficient for interpretive estimates.

#### Construct validity

Construct validity is the degree to which a scale captures the construct it is purported to measure, that is typically evaluated by showing associations with other concurrently assessed measures of that construct or similar constructs, and a lack of association with other concurrently assessed measures of different or dissimilar constructs. One approach to evaluating the construct validity of a target instrument is to assess the degree to which variations in scores on that instrument are concordant with variations in scores from instruments assessing putatively related constructs relative to instruments assessing putatively unrelated constructs. In this study, we used TICS to measure a construct (cognitive dysfunction) that is conceptually related to the MRC-PDRS, and in particular cognitive domains captured by the MRC-PDRS – a ‘cognitive component’ comprised of the three domains on the MRC-PDRS that capture verbal report, memory/orientation, and judgment/problem solving – while we used the NPI-Q to measure a construct (neuropsychiatric symptoms) that is not conceptually related to outcomes captured by the MRC-PDRS.

We examined construct validity using three approaches. The first two approaches, convergent and divergent validity, were evaluated based on the strength and direction of correlations between the target measure(s) and other concurrently administered measures of conceptually related or conceptually unrelated constructs, respectively. Convergent validity was evaluated using Pearson correlation coefficients at each visit for MRC-PDRS total scores and the MRC-PDRS cognitive component (sum of the three cognitive domains) with TICS scores. Divergent validity was evaluated using Pearson correlation coefficients at each visit between MRC-PDRS total scores and NPI-Q scores. Evidence supporting acceptable convergent validity are correlation coefficient values ≥|0.40|, with correlations <|0.30| considered as evidence for divergent validity [24]. Both convergent and divergent validity were evaluated at each of the first four visits for each patient.

The third approach for evaluating the construct validity of MRC-PDRS scores was known-groups discriminant validity, which assesses differences in these scores among subgroups of patients classified by a measure of a conceptually related construct for which participants are known to vary in this construct, in this case classification of TICS scores. Mean MDRC-PDRS total and cognitive component scores were compared across cognitive impairment severity subgroups defined by TICS scores[20]. Given the small number of patients with no cognitive impairment in the TAPCJD dataset as defined by the TICS (i.e., only 4 patients at the initial visit, with even fewer at later visits), we compared MRC-PDRS scores across 3 cognitive severity subgroups, in which we combined patients with no cognitive impairment with patients who had ambiguous cognitive impairment into a single group (i.e., those with a TICS score ≥ 26), along with mildly impaired and moderately or severely impaired subgroups. Differences in mean MRC-PDRS scores across all subgroups were tested using one-way analysis of variance (ANOVA) models, while magnitudes of pairwise differences among the three subgroups were examined using Cohen’s *d* effect sizes for standardized mean differences, interpreted using guidelines suggested by Cohen: *d* = 0.20, small effect; *d* = 0.50, medium sized-effect; and *d* = 0.80, large effect [25]. Known-groups discriminant validity was evaluated at the first two visits for each patient, to ensure that all subgroups defined by TICS scores included no fewer than 5 patients.

#### Responsiveness

The responsiveness of an instrument, or its ability to detect change, can be estimated by the degree to which changes in a target measure concord with changes in other measures that capture the same or similar latent construct it is purported to measure. In the current analysis, the responsiveness of MRC-PDRS total and cognitive component scores was examined by evaluating the magnitude and direction of Pearson correlations between changes in these scores with changes in TICS scores from the initial visit (Visit 1) to the second, third, and fourth visits for each patient. A Pearson correlation coefficient ≥|0.30| between changes in these measures was considered as supportive of adequate responsiveness [26].

#### Distribution-based estimates of minimally important change thresholds for MRC-PDRS total score

Distribution-based approaches for estimating MIC thresholds use the target measure’s variability, and in some instances its reliability, to estimate a threshold past which a magnitude of change exceeds what could be accounted for by random measurement error. In this analysis, MIC thresholds were estimated for change in MRC-PDRS total scores using three types of distribution statistics: effect size (ES), standardized response mean (SRM), and the reliable change index (RCI).

For ES, the mean change is set to 0.5, which is the value that, according to Cohen’s interpretation guidelines, corresponds to a medium-sized difference [22]. This value is then multiplied by the standard deviation (SD) of the score at the initial visit (SD_V1_), yielding the equivalent of ½ SD, which past studies have shown to concord closely to estimates yielded from anchor-based approaches [27, 28].

SRM, like ES, is based on standardized mean, although in this case for change (within-person) rather than differences (between-groups). For SRM, the mean change is also set to 0.5, but this value is then multiplied by the SD for change from one visit to another, rather than for the initial visit. The current study calculated two SRM values, with one estimate based on SD for mean change from Visit 1 to Visit 2 (SD_V1V2_) and the other estimate based on SD for mean change from Visit 1 to Visit 3 (SD_V1V3_).

RCI is calculated using both the magnitude of change over time in individuals’ scores on a measure, as well as the variability and reliability of that measure, with the latter two properties captured via the standard error of measurement (SEM) of an instrument. Peipert, Cella, and Hays (2024) have recently argued that RCI threshold as the ideal lower bound threshold of individual change [29]. SEM is calculated by multiplying SD_V1_ by the square-root of one minus the measure’s reliability. Jacobson and Truax’s (1991) RCI is calculated as the change in mean score on a measure at two assessments (X_1_ – X_2_) divided by SEM, with SEM having a multiplier of the square root of 2: (X_1_ – X_2_)/($\sqrt 2$ * SEM) [30]. For the current analysis, we calculated SEM using the internal-consistency reliability estimate of MRC-PDRS calculated using McDonald’s ω at Visit 1 in the current (NPDPSC) dataset. As for SRM, we calculated two RCI values, with one estimate based on the mean change from Visit 1 to Visit 2 (X_V1_ – X_V2_) and the other estimate based on the mean change from Visit 1 to Visit 3 (X_V1_ – X_V3_).

## Results

### Population

Data from 101 participants from the TAPCJD study were included in the analyses (Table 1). MRC-PDRS scores were obtained for all 101 participants at Visit 1 through Visit 4 (approximately 4 months after Visit 1). No statistically significant differences were found in gender, age, or race and ethnicity among the participants. The mean age of participants at the onset of illness was 65 years. The median illness duration, defined as the period between initial symptom onset (which occurred before the first visit) until death, was 11 months. A mean of 4.4 months (median of 2.5 months) elapsed between the initial visit and death.

**Table 1:** Demographic information of participants enrolled in the study (n = 101)

Mean age at onset (years)	64.6 ± 9.6 [22–88]
Median age at onset (years)	65.0 [60–69]
Mean time from first visit to death (months)	4.4 ± 5.5 [0–30]
Median time from first visit to death (months)	2.5 [0–6]
Median illness duration (months)	11.0 [7–16]
Female	48 (48)
Race	
White	91 (90.0)
Hispanic	4 (4.0)
African American	4 (4.0)
Asian	2 (2.0)
Cerebrospinal fluid results^1^	
Positive 14-3-3 protein (n = 86)	76 (88.3)
Mean tau level, pg/mL (*n* = 87)	4979.1 ± 5327.7 [0–27,111]
Positive RT-QuIC (n = 87)	75 (86.2)
Prion Disease Diagnosis (n = 88)^2^	
Sporadic Creutzfeldt-Jakob disease (sCJD)	76 (86)
Genetic Prion Disease (gPrD)	12 (14)

Values listed in the tables are means and SD with [ranges]/ medians [IQR] or numbers (%)

*Abbreviations*: sCJD, Sporadic Creutzfeldt-Jakob disease; gPrD, Genetic Prion Disease; ^1^Cerebrospinal fluid tests were performed at the National Prion Disease Pathology Surveillance Center. ^2^Diagnostic criteria based on Parchi et al [7]

The final diagnosis was confirmed through autopsy for 88 (87%) participants. Among those with confirmed diagnoses, sporadic CJD (sCJD) was the most common diagnosis (*n* = 76, 86.4%). Within sCJD, the MV2 variant of sCJD was the predominant subtype, observed in 20 participants (22.7%). 13 participants (13%) did not receive an autopsy. Further categorization by subtype of sCJD and genetic CJD (gCJD) of the participants who went to autopsy, and additional demographic information is illustrated in the supplemental material.

**Fig. 1:**
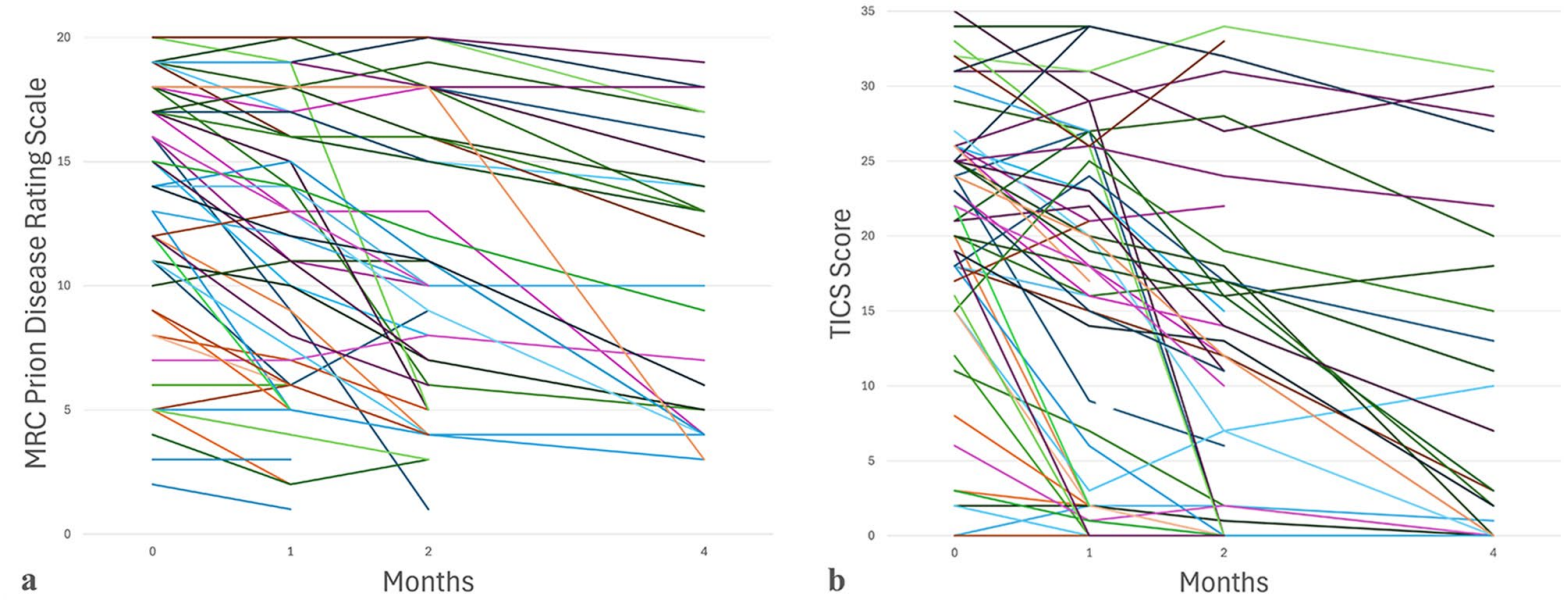
Spaghetti plots of MRC-PDRS (**a**) and TICS (**b**) scores from the initial visit to Visit 3 four months following the initial visit demonstrating longitudinal progressive disease severity and cognitive decline, respectively

The mean (SD) MRC-PDRS total score at the initial visit was 11.6 (5.9), with scores observed across the full range, from 0 to 20 (Table 2) and with most patients declining at subsequent visits, unless the patient dropped out of the study or died before decline was observed (Fig. 1a).

The mean (SD) TICS score at Visit 1 was 13.3 (11.7), with observed scores ranging from 0 to 35, demonstrating wide initial variability (Table 2), and with rapid decline following in subsequent visits (Fig 1b).

**Table 2:** MRC-PDRS and TICS results demonstrating longitudinal progression in disease severity and cognitive decline, respectively

Scale	N	Mean score
MRC-PDRS		
Visit 1	98	11.6 ± 5.9 [0–20]
Visit 2	60	12.4 ± 5.8 [1–20]
Visit 3	48	11.4 ± 6.0 [1–20]
Visit 4	28	10.8 ± 5.7 [3–19]
TICS		
Visit 1	97	13.3 ± 11.7 [0–35]
Visit 2	60	14.2 ± 11.7 [0–34]
Visit 3	48	11.1 ± 11.0 [0–34]
Visit 4	27	9.0 ± 10.9 [0–31]

Values are reported as means and SD with [ranges]

*Abbreviations:* MRC-PDRS, Medical Research Council Prion Disease Rating Scale; SD, standard deviation; TICS, Telephone Interview for Cognitive Status

**Fig. 2:**
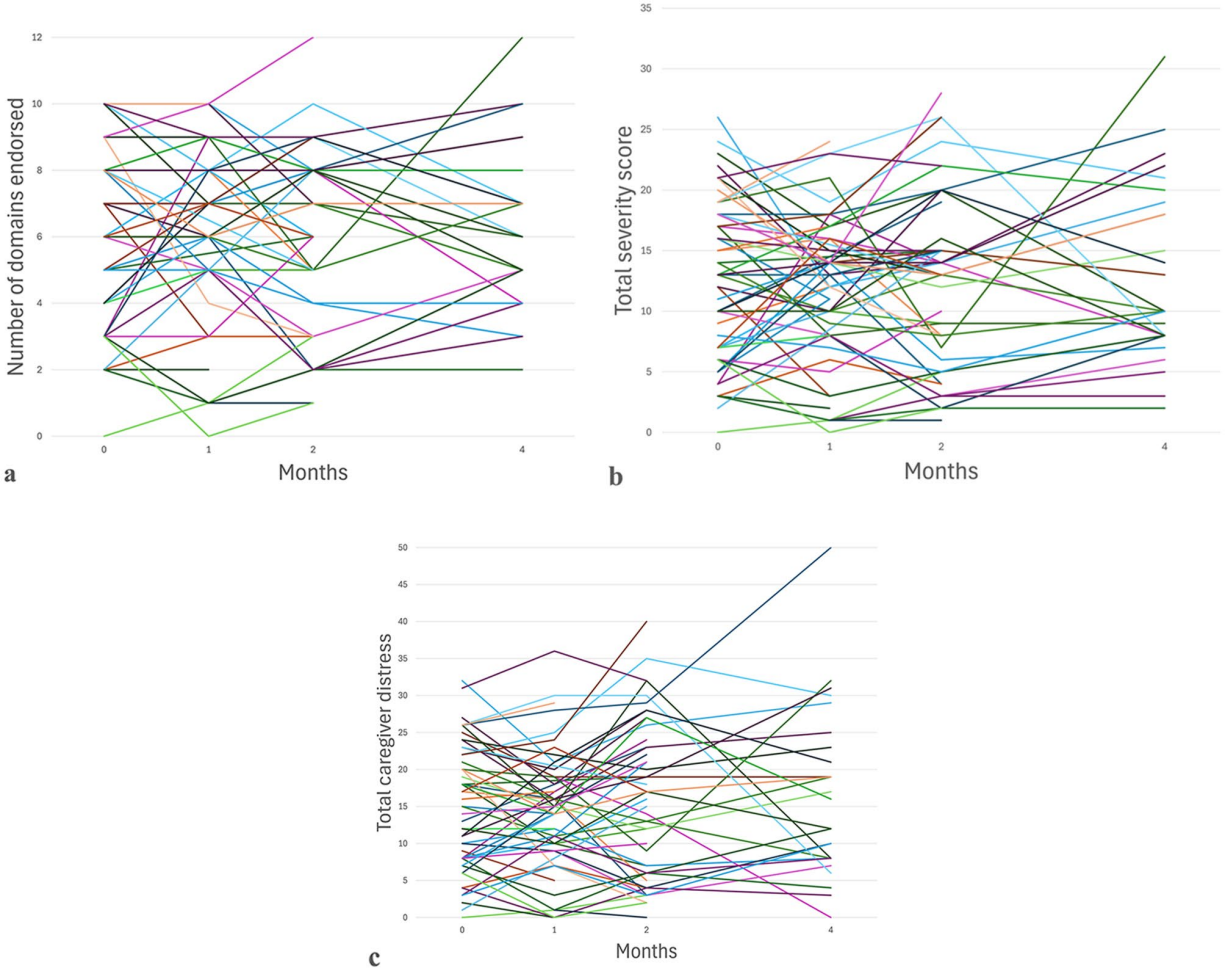
Spaghetti plots of NPI-Q scores: domains endorsed (**a**), severity score (**b**), caregiver distress score (**c**) from the initial visit to Visit 3 four months following the initial visit demonstrating variability of neuropsychiatric symptoms between participants and between study visits

Mean NPI-Q scores are shown in Table 3. NPI-Q domains endorsed, severity, and caregivers distress scores and their change over time varied widely across patients at the initial visit and later visits, as shown in Fig. 2a, 2b, and 2c, respectively, varied across the participants in the study.

**Table 3:** NPI-Q results demonstrating variability in neuropsychiatric symptoms between study visits

Visit	N	Mean number of symptoms endorsed	Mean severity score	Mean caregiver distress score
1	96	6.5 ± 2.7 [0–12]	14.3 ± 7.4 [0–34]	17.3 ± 10.3 [0–44]
2	56	6.1 ± 2.6 [0–10]	11.7 ± 5.9 [0–24]	13.8 ± 7.9 [0–36]
3	50	6.1 ± 2.7 [1–12]	12.3 ± 7.1 [0–28]	15.9 ± 10.3 [0–40]
4	28	6.4 ± 2.5 [2–12]	13.0 ± 7.3 [0–31]	16.1 ± [0–50]

Values are reported as means and SD with [ranges]

*Abbreviations:* NPI-Q, Neuropsychiatric Inventory Questionnaire; SD, standard deviation

### Internal consistency

Cronbach’s α and McDonald’s ω estimates at Visits 1 through 4 are presented in Table 4. Estimates for both statistics at all visits exceed 0.90, supporting excellent internal consistency reliability for MRC-PDRS total score.

**Table 4:** Internal Consistency – Cronbach’s α and McDonald’s ω for MRC-PDRS Total Score demonstrating excellent internal consistency in MRC-PDRS score across study visits

Visit	N	Cronbach’s α	McDonald’s ω
1	98	0.94	0.94
2	60	0.94	0.95
3	48	0.95	0.96
4	28	0.95	0.95

### Convergent validity

Pearson correlations for MRC-PDRS total and cognitive component scores with concurrently assessed TICS scores at Visits 1–4 are reported in Table 5. All correlations are strong (≥0.74), well exceeding the criterion of 0.40 in support of convergent validity. The magnitude of correlations with TICS were similar for both the MRC-PDRS total and cognitive component scores.

**Table 5:** Convergent Validity – Pearson Correlations between MRC-PDRS Total Scores and Concurrent TICS demonstrating good convergent validity between a test of general cognition (TICS) and the cognitive component of the MRC-PDRS as well as its overall score

Visit	N	MRC-PDRS Total (*r*)	MRC-PDRS Cognitive Component (*r*)
1	95	0.77	0.83
2	59	0.81	0.78
3	47	0.86	0.89
4	27	0.80	0.74

### Divergent validity

As shown in Table 6, Pearson correlations between the MRC-PDRS and all three subscales of the NPI-Q were all weak, with none exceeding an absolute magnitude of 0.20, supporting the divergent validity of the MRC-PDRS.

**Table 6:** Divergent Validity – Pearson Correlations between MRC-PDRS and NPI–Q demonstrating that disease severity as measured by MRC-PDRS is not associated with neuropsychiatric symptoms as measured by NPI-Q

Visit	N	NPI-Q Symptoms (*r*)	NPI-Q Severity (*r*)	NPI-Q Caregiver Distress (*r*)
1	96	−0.07	−0.12	−0.10
2	56	0.03	−0.05	0.00
3	49	0.05	−0.07	0.02
4	27	0.12	0.02	0.20

### Known-groups discriminant validity

The results from comparison of MRC-PDRS total and cognitive components across the three categories of TICS scores are reported in Tables 7 and 8, respectively. For both target measures at both visits, omnibus ANOVA models found statistically significant differences in MRC-PDRS scores across all three severity levels (all *p* < 0.001). Additionally, effect sizes for pairwise differences among the three severity categories yielded mostly large effects across scores, reflecting stepwise decreases in MRC-PDRS scores as a function of increased severity of cognitive impairment based on classifications using TICS scores. Large to very-large effect sizes were observed for differences between patients classified with no or ambiguous cognitive impairment compared to patients classified with mild or moderate/severe cognitive impairment (*d*’s ranging from 0.93 to 2.01), while moderate to very-large effect sizes were observed for differences between patients classified with mild compared to moderate or severe cognitive impairment (*d*’s ranging from 0.64 to 1.26). Similar to findings from convergent validity analyses, the magnitude of associations between differences in MRC-PDRS total and cognitive component scores with differences in TICS scores were generally comparable.

**Table 7:** Known-Groups Discriminant Validity – MRC-PDRS Total Score by TICS Categories (no/ambiguous impairment, mild impairment, and moderate/severe impairment) demonstrating strong relationships between MRC-PDRS overall score and degree of cognitive impairment as measured by TICS

***Visit***	No/Ambiguous Impairment			Mild Impairment			Moderate/Severe Impairment			p*	No/Ambiguous vs. Mild	No/Ambiguous vs. Moderate/Severe	Mild vs. Moderate/Severe
	***N***	***Mean***	***SD***	***N***	***Mean***	***SD***	***N***	***Mean***	***SD***		***d***	***d***	***d***
1	16	17.9	1.6	16	15.3	3.6	63	8.9	5.4	<0.001	0.93	1.84	1.26
2	14	18.1	3.0	7	13.9	3.4	38	10.0	5.5	<0.001	1.34	1.63	0.74

* Based on a one-way ANOVA model with TICS category as a fixed factor

*Abbreviations:* MRC-PDRS, Medical Research Council Prion Disease Rating Scale; SD, standard deviation; TICS, Telephone Interview for Cognitive Status

**Table 8:** Known-Groups Discriminant Validity – MRC-PDRS Cognitive Component Score by TICS Categories (no/ambiguous impairment, mild impairment, and moderate/severe impairment) demonstrating strong relationships between the cognitive component of the MRC-PDRS and degree of cognitive impairment as measured by TICS

***Visit***	No/Ambiguous Impairment			Mild Impairment			Moderate/Severe Impairment			p*	No/Ambiguous vs. Mild	No/Ambiguous vs. Moderate/Severe	Mild vs. Moderate/Severe
	***N***	***Mean***	***SD***	***N***	***Mean***	***SD***	***N***	***Mean***	***SD***		***d***	***d***	***d***
1	16	7.3	0.7	16	5.9	3.6	63	3.9	1.8	<0.001	1.61	2.01	1.22
2	14	6.9	1.2	7	5.9	3.4	38	4.5	1.8	<0.001	1.19	1.44	0.64

* Based on a one-way ANOVA model with TICS category as a fixed factor

*Abbreviations:* MRC-PDRS, Medical Research Council Prion Disease Rating Scale; SD, standard deviation; TICS, Telephone Interview for Cognitive Status

### Responsiveness

Table 9 reports results from evaluation of the responsiveness of MRC-PDRS, as Pearson correlations between changes in MRC-PDRS total and cognitive composite scores with changes in TICS scores from Visit 2 through 4. All correlations for both target measures exceeded the criterion value of 0.30 across all visits, while for the longer intervals (i.e., from Visit 1 to Visits 3 and 4), all *r* > 0.65, indicating strong correlations. These results for both sets of MRC-PDRS scores show that decreases (worsening) in MRC-PDRS scores were associated with decreases (worsening) in TICS scores over time, supporting the responsiveness of both target measures.

**Table 9:** Responsiveness – Pearson Correlations with Changes from Initial Visit in TICS demonstrating good responsiveness of the cognitive component and overall MRC-PDRS score and cognitive decline as detected by TICS across study visits

Visit	N	MRC-PDRS Total (*r*)	MRC-PDRS Cognitive Component (*r*)
2	58	0.41	0.37
3	46	0.71	0.72
4	27	0.67	0.66

### Distribution-based estimates of minimally important change thresholds for MRC-PDRS total score

Distribution-based estimates of MIC thresholds are reported in Table 10. While there is some expected variation among estimates, they range from approximately 1 to 3 points, supporting a MIC threshold of this range of magnitude.

**Table 10:** Distribution-Based Estimates of Minimally Important Change (MIC) Thresholds for MRC-PDRS Total Score demonstrating the MRC-PDRS ability to detect MIC with small changes in its overall score

Visit	N	ES (0.5 SD)	RCI_V1-V2_	RCI_V1-V3_	SRM_V1-V2_	SRM_V1-V3_
1	98	2.9	0.7	1.7	1.1	2.0

*Abbreviations:* ES, effect size; ICR, internal consistency; IRR, inter-rater reliability; RCI, reliable change index; SD, standard deviation; SRM, standardized response mean; V, visit

## Discussion

This analysis of data from the TAPCJD study focused on assessing measurement properties and interpretation guidelines for the MRC-PDRS. The findings of our study provide strong evidence supporting the internal consistency, construct validity, and responsiveness of the MRC-PDRS in patients with prion disease. Further, distribution-based estimates provide preliminary support for interpreting a change of 1–3 points in MRC-PDRS total score as reflecting minimally significant change in a patient’s disease status.

Most statistical estimates far exceeded criteria used to support adequate psychometric properties. Correlations between MRC-PDRS scores and TICS scores were generally quite strong, as were standardized mean differences in MRC-PDRS across severity of cognitive impairment defined by TICS scores, and most correlations for changes in MRC-PDRS scores with changes in TICS scores across visits.

Even though the TICS is a measure of cognitive function, the association of the MRC-PDRS cognitive component, which includes the items on the measure specifically capturing cognitive-related functions (expressive language, memory/orientation, and judgment/problem solving), was surprisingly not stronger than that between the TICS and the MRC-PDRS total score. That is, given the greater conceptual overlap of the cognitive component with the content of the TICS, we had expected to find a stronger concordance with this subset of MRC-PDRS domains than for the full scale. The comparability of the total scale with the cognitive component in this respect confirms the assumptions of this Rasch derived scale of the blindness to item content, such that the change of 1 point on any domain is equivalent to the same magnitude of change on any other domain. Thus, any change on MRC-PDRS scores, whether related to specific changes in cognitive function or other aspects of functioning, such as motor function, were seemingly equally sensitive to predict changes in scores on a measure of cognitive function. This quality is especially important in prion disease, in which symptoms and progression vary by sCJD molecular subtype and by mutation. Even though symptom progression may differ between individuals, the MRC-PDRS detects a fairly consistent progression of disease.

This study has several limitations. First, while the sample size at the initial visit was relatively large, particularly for such a rare condition, the sample size at later visits substantially decreased. This is not surprising given the very fast progression of prion disease. A second limitation of the study was the limited range of criterion measures, with a heavy reliance on the TICS for evaluation of construct validity and responsiveness. Additionally, the prion disease population in this study, with an overrepresentation of sCJD MV2 and underrepresentation of sCJD MM1, is not representative of the known human prion disease subtype population [31]. This is likely due to the rapid progression of some of the more common sCJD subtypes (e.g., MM1, VV2), precluding their entry into such a longitudinal study. Although our study sample differs from the general human prion disease population, it is likely to closely resemble those that would be most likely to be diagnosed and identified for a potential treatment. Additionally, participants varied in disease stage at each visit, suggesting generalizability of these findings throughout stages of clinical severity.

Lastly, another limitation of this study was the reliance on distribution-based approaches for estimating thresholds indicating interpretation guidelines for change in disease progression as captured by changes in MRC-PDRS scores. It has been well established that anchor-based methods are generally superior to distribution-based methods for generating such thresholds, since the former capture change on a known “gold-standard” measure that provides a clear and straightforward indication of a meaningful change in a patient’s health status, while the latter are based solely on statistical properties of the measure [32]. Since this study did not include appropriate anchor measures, only distribution-based methods were possible. As such, the estimates for MIC thresholds of MRC-PDRS scores reported from this analysis should be regarded as preliminary, and future research using datasets where such anchors are available should be used to estimate thresholds of clinically meaningful change in the MRC-PDRS.

The results of this study may also enhance clinical care for patients with prion disease. Tests demonstrating strong responsiveness and validity, as the MRC-PDRS demonstrated in this study, provide clinicians with a more reliable tool for monitoring disease progression, which is crucial for rapidly progressive conditions like sCJD. MRC-PDRS scores obtained in clinical settings can assist clinicians in prognostication, formulating more effective treatment options and serve as a valuable communication tool to help patients and their families understand the disease and the rationale behind management strategies. Finally, responsiveness is essential for evaluating potential benefits of future treatment options during clinical trials. Survival time is an important outcome measure but does not convey quality of life information or functional abilities. Additionally, human prion diseases are clinically heterogeneous and using an outcome measure that appropriately measures overall disease progression is imperative [2]. This study found the MRC-PDRS to be a robust measure of determining disease severity throughout the duration of disease, a feature that is unprecedented in the field of prion disease. Moreover, its ability to detect MIC with small changes in overall score positions the MRC-PDRS to be useful in prognosticating and determining potential effects of clinical treatment trials. This study further validates that the MRC-PDRS could be used as a primary endpoint measure in a clinical trial that would address quality of life and disability concerns as well as mitigate issues with variability of survival times seen in different prion disease subtypes.

## Conclusion

Our findings in this study indicate that the MRC-PDRS is a reliable, valid, and responsive tool for capturing functional disabilities in patients with prion disease. This study, utilizing data from the TAPCJD and comparing metrics from MRC-PDRS, TICS, and NPI-Q, provides strong preliminary evidence supporting the MRC-PDRS’s efficacy in measuring disease progression in patients with human prion disease. Furthermore, analyses were conducted to estimate preliminary thresholds for identifying interpretation guidelines for changes in MRC-PDRS scores. These findings can inform clinical trials aimed at evaluating the benefits of treatments for patients with human prion disease and inform practice of clinical care of patients with this disease.

## Electronic supplementary material

Below is the link to the electronic supplementary material.

Supplementary Material 1

## Data Availability

The data and analyses that support this study are available from the corresponding author upon request.

## Abbreviations

ANOVA: Analysis of variance
ES: Effect size
gCJD: Genetic Creutzfeldt-Jakob Disease
gPrD: Genetic Prion Diseases
GSS: Gerstmann-Strausler-Scheinker Syndrome
ICR: Internal consistency
IQR: Interquartile range
IRR: Inter-rater reliability
MIC: Minimally important change
MRC: Medical Research Council
MRC-PDRS: Medical Research Council-Prion Disease Rating Scale
NPDPSC: National Prion Disease Pathology Surveillance Center
NPI-Q: Neuropsychiatric Inventory-Questionnaire
PrPc: Cellular Prion Protein
PrPSc: Pathological Cellular Prion Protein
RCI: Reliable change index
RT-QuIC: Real-Time Quaking-Induced Conversion
sCJD: Sporadic Creutzfeldt-Jakob Disease
SD: Standard deviation
SEM: Standard error of measurement
SRM: Standardized response mean
TAPCJD: Telemedicine Assessment Program for Creutzfeldt-Jakob Disease
TICS: Telephone Interview for Cognitive Status
V: Visit
