# Supplementary material for: Internal consistency, construct validity, and responsiveness of the MRC Prion Disease Rating Scale
Source: J Patient Rep Outcomes. 2025 May 6;9:49. doi: 10.1186/s41687-025-00884-3 (PMC12055732; doi:10.1186/s41687-025-00884-3)
Supplement: Supplementary file 1 — Supplementary Material 1 [file 41687_2025_884_MOESM1_ESM.docx]

**Supplemental Material**

Final Autopsy-confirmed Diagnosis

| Final Autopsy-confirmed Diagnosis (n = 88)^1^ | |
| --- | --- |
| sCJD, MV1 | 5 (5.6) |
| sCJD, MV2 | 20 (22.7) |
| sCJD, MV1-2 | 16 (18.2) |
| sCJD, MM1-2 | 10 (11.4) |
| sCJD, MM1 | 6 (6.8) |
| sCJD, MM2 | 6 (6.8) |
| sCJD VV1-2 | 3 (3.4) |
| sCJD, VV1 | 2 (2.3) |
| sCJD, VV2 | 6 (6.8) |
| sCJD, VPSPr | 2 (2.3) |
| gCJD (D178N-129V) | 4 (4.5) |
| gCJD (E200K-129M) | 2 (2.3) |
| gCJD (V189I-129M) | 1 (1.1) |
| gCJD (R208H-129V) | 1 (1.1) |
| gCJD (E200K-129V) | 1 (1.1) |
| GSS (A117V-129V) | 1 (1.1) |
| GSS (P102L-129M) | 2 (1.1) |
| *Values listed in the tables are numbers (%). Abbreviations: sCJD, Sporadic Creutzfeldt-Jakob disease; gCJD, Genetic Creutzfeldt-Jakob disease; GSS, Gerstmann-Straussler-Scheinker Syndrome. ^1^Diagnostic criteria based on Parchi et al.*^7^ | |
